# The N terminus of Ascl1 underlies differing proneural activity of mouse and *Xenopus* Ascl1 proteins

**DOI:** 10.12688/wellcomeopenres.14842.1

**Published:** 2018-09-26

**Authors:** Laura J.A. Hardwick, Anna Philpott

**Affiliations:** 1 Wellcome-MRC Cambridge Stem Cell Institute, University of Cambridge, Cambridge, CB2 1QR, UK; 2 Department of Oncology, University of Cambridge, Cambridge, CB2 0XZ, UK; 3 Peterhouse, University of Cambridge, Cambridge, CB2 1RD, UK

**Keywords:** Ascl1, neurogenesis, proneural, Xenopus, bHLH.

## Abstract

The proneural basic-helix-loop-helix (bHLH) transcription factor Ascl1 is a master regulator of neurogenesis in both central and peripheral nervous systems *in vivo,* and is a central driver of neuronal reprogramming *in vitro*. Over the last three decades, assaying primary neuron formation in *Xenopus* embryos in response to transcription factor overexpression has contributed to our understanding of the roles and regulation of proneural proteins like Ascl1, with homologues from different species usually exhibiting similar functional effects. Here we demonstrate that the mouse Ascl1 protein is twice as active as the *Xenopus* protein in inducing neural-β-tubulin expression in *Xenopus* embryos, despite there being little difference in protein accumulation or ability to undergo phosphorylation, two properties known to influence Ascl1 function. This superior activity of the mouse compared to the *Xenopus* protein is dependent on the presence of the non-conserved N terminal region of the protein, and indicates species-specific regulation that may necessitate care when interpreting results in cross-species experiments.

## Introduction

Proneural basic-helix-loop-helix (bHLH) transcription factors have conserved roles from *Drosophila* to vertebrates, acting in cascades to drive and coordinate the multiple stages of neurogenesis ^1^. Vertebrate homologues of the *Drosophila achaete-scute complex* include mammalian Ascl1 (mash1/mAscl1) ^2^, and *Xenopus* Ascl1 (Xash1/xAscl1) ^3^. In mammals, Ascl1 is involved in development of GABAergic neurons in the ventral telencephalon and dorsal spine ^4^, mesencephalic dopaminergic neurons ^5^, rhombencephalic serotonergic neurons ^6^ and central and peripheral noradrenergic neurons ^7^. In parallel, *Xenopus* Ascl1 is expressed pre and post-metamorphically in the ventricular zone of the pros-, mes-, and rhombencephalon ^3^, with a distinct role in GABAergic fate in the retina ^8^, and in noradrenergic identity in antero-ventral neural precursors ^9^. 

Development of *Xenopus* primary neurons expressing neural-β-tubulin has provided a simple assay for investigating proneural protein activity and regulation *in vivo*, for example ^10– 12^. Proneural gene homologues from different species are often used interchangeably due to the striking conservation of expression and function as outlined above. Ascl1 is also a key cellular reprogramming factor in the regenerative medicine field ^13^ and its molecular mechanisms of action are a current area of study where species-specific phenomena may be critical for selection of appropriate model systems. We present a short study to compare the activity of mouse and *Xenopus* Ascl1 proteins in driving neurogenesis in *Xenopus* embryos. Surprisingly, we observe a marked difference in potency between the mouse and *Xenopus* Ascl1 homologues, and show that the differences in potency map to the non-conserved residues in the N terminus of the protein.

## Methods

### Animal care

All work has been carried out under UK Home Office Licence and has passed an Institutional ethical review committee assessment at the University of Cambridge.

### Plasmids and constructs

The coding regions of mouse *Ascl1* (Genbank accession number NM008553) and *Xenopus Ascl1* (Genbank accession number NM001085778) were cloned into pCS2+ vectors between EcoR1 and Xho1 or BamH1 and Xho1 sites respectively, adding a single HA tag at 3’ end. The chimeric N-m/bHC-xAscl1 construct was generated by double digest of both plasmids with BamH1 and Bgl1. The N terminal fragment of the mouse gene was purified as the insert and the bHLH and C terminus of the *Xenopus* gene in pCS2+ was used as the vector for ligation. The NTdelxAscl1 construct was generated by PCR amplification of the conserved N terminal region, bHLH and C terminus of the *Xenopus* gene, (therefore removing the non-conserved 5’region of the N terminus) using primers

[5’ GATCGGATCCACCATGAAGAGGCAACGCTCGG] and

[3’ GATCCTCGAGTCAGAACCAAGTGGTGAAGTC] with cloning into pCS2+ between BamH1 and Xho1. Nucleotide and protein sequence alignments were conducted with ClustalW software ^14^.

### *Xenopus laevis* embryo manipulation

All efforts are made to ameliorate suffering to any animal. For example, the colony of approximately 80 *X. laevis* females are housed and cared for by a dedicated team of animal technicians operating under Home Office Licence. Each experiment requires eggs from 2 or 3 females (depending on N = 2 or 3) and females are used on rotation within the colony with at least a 3-month rest period after laying. A single male frog is sacrificed under humane conditions and Home Office Licence to provide testes for at least 16 experiments. Embryos obtained from fertilised eggs are used for the experiments and development is stopped 48 hours post fertilisation when embryos reach late neurula stage and prior to formation of tadpoles.

Thus, *X. laevis* eggs were obtained by standard hormone methods of induction, and subsequently fertilised *in vitro*. pCS2+ constructs were linearised and capped mRNA was transcribed *in vitro* using the SP6 mMessage mMachine ^®^ kit (Ambion). Embryos were injected unilaterally at the two cell stage with mRNA as indicated in the text, with GFP (for qPCR) or β-gal (ISH) as lineage tracers. Embryos were cultured at 18°C in Ficol solution and staged according to ^15^. At stage 18, embryos were either snap-frozen for qPCR analysis or fixed in MEMFA for 90 minutes, washed twice in PBS, followed by PBS supplemented with 2mM MgCl _2_ and embryos stained in 1 mg/ml X-gal in X-gal mixer. For recipes see ^12^. Embryos were again washed twice in PBS before dehydration and storage in methanol.

### Whole mount *in situ* hybridisation (ISH)

Dig-oxigenin-labelled anti-sense probes were synthesised from the following plasmids: *X. laevis neural-β-tubulin* ^16^ and *X. laevis xMyt1* ^17^. Whole mount ISH was performed as described in 12 and embryos were scored for the extent and pattern of marker expression as described in data analysis.

### Quantitative real-time PCR (qPCR)

GFP expression was used to confirm successful injection and samples of four embryos were snap frozen. Whole embryo RNA was extracted using the RNeasy ^®^ Mini kit (Qiagen) and template cDNAs synthesised with the QuantiTect ^®^ Reverse Transcription Kit (Qiagen). qPCR was performed using the Quantifast ^®^ SYBR Green PCR kit (Qiagen) in a LightCycler ^®^ 480 (Roche). Thermal cycling conditions: 95°C for 5 minutes, then 45 cycles of 95°C for 10s, 60°C for 10s and 72°C for 20s. EF1α reference gene (Genbank accession NM001087442): Forward, CACCATGAAGCCCTTACTGAG; Reverse, TGATAACCTGTGCGGTAAATG. N-β-Tubulin target gene (Genbank accession NM001086064): Forward, TGGATTTGGAACCAGGCA; Reverse, GCTCAGCTCCTTCGGTGTA.

### Western blotting

For western blot analysis, 12 embryos were snap frozen at stage 12.5 and whole embryo protein was extracted as described in 12. For detection of phospho-status, samples were incubated in the presence or absence of lambda protein phosphatase (NEB) for 1 hour at 30°C. 60µg total protein was loaded on to pre-cast BioRad Criterion ^™^ TGX 18% gels in Tris-Glycine buffer. Primary antibodies were used at 1:2000 dilution for at least 1 hour at room temperature (tubulin) or at 4°C overnight (HA): Rat HRP-conjugated anti-HA clone 3F-10 antibody (Roche; 12013819001) and mouse anti-α-tubulin clone B-5-1-2 antibody (Sigma; T5618). Anti-tubulin antibody was detected with a sheep HRP-conjugated anti-mouse antibody at 1:10000 dilution (GE Healthcare; NA931V).

### Data analysis

For ISH data, embryos were scored for the extent and pattern of gene expression on the injected side relative to uninjected side and to uninjected embryos. Scores were assigned as 0, no difference; +1, mild increase in expression within the neural tube with or without occasional ectopic expression on the injected side; +2, moderate increase with ectopic expression occurring in patches on the injected side and sometimes bilaterally; +3, substantial increase with extensive ectopic expression in a more homogenous pattern on the injected side and sometimes bilaterally. Experiments were conducted in independent duplicate in different batches of eggs and the N numbers refer to the range of total numbers of embryos in each injection category.

For qPCR data, mRNA expression was normalised to expression of reference gene EF1α and mRNA levels in the injected embryos were calculated relative to stage-matched uninjected controls. Mean values are plotted and error bars show the standard error of the mean from three independent experiments. Statistical significance was calculated by a paired two-tailed student T test; NS = not significant; * = p< 0.05; ** = p< 0.025; *** = p< 0.0125. Western blots show two independent experiments. For protein quantification, Image J software was used as described in ^12^.

## Results

### Mouse Ascl1 is more active than *Xenopus* Ascl1 in the *Xenopus* primary neurogenesis assay

*Xenopus* primary neurons endogenously develop in the trigeminal ganglia and three bilateral stripes on the neural plate ^18^, but over-expression of proneural proteins by micro-injection of mRNA can lead to expansion of the endogenous domains and induction of neural-β-tubulin expression across the lateral and ventral epidermis ^12^. In order to directly compare the effects of over-expression of the mouse and *Xenopus* Ascl1 proteins, equivalent plasmids were constructed with the coding region of each wild-type gene placed directly after the SP6 promoter and with a single C terminal HA tag.

**Figure 1.**
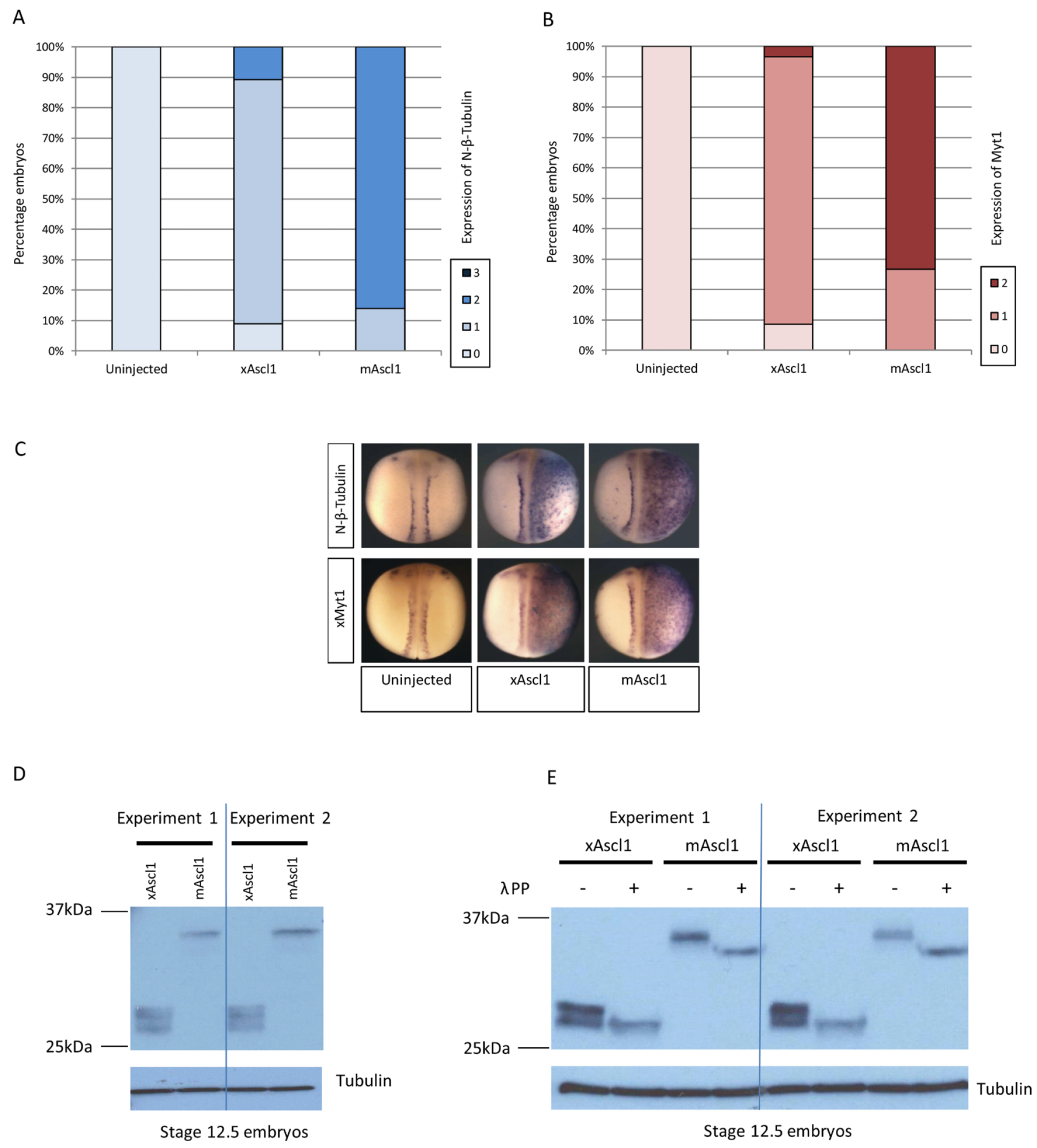
mAscl1 is more active than xAscl1 despite no difference in protein stability. ( **A**– **C**) Two cell stage embryos were unilaterally injected with 100pg of mRNA encoding either xAscl1 or mAscl1. At stage 18, embryos were assayed by *in situ* hybridisation (ISH) and scored for gene expression relative to uninjected control embryos. ( **A**) N-β-tubulin expression [N=50-78 embryos per category from two experiments]. ( **B**) Myt1 expression [N=58-63 embryos from two experiments]. ( **C**) Representative images of embryos with injected side to the right, stained with pale blue β-gal tracer. Injection of equal amounts of mRNA results in greater gene upregulation by mAscl1 than xAscl1. ( **D**) Western blot analysis of stage 12.5 whole embryo extracts from two independent experiments, over-expressing 200pg of each HA-tagged construct and detected with anti-HA antibody; tubulin as a loading control. There are no significant differences in protein expression between the two constructs. ( **E**) Whole embryo extracts from ( **C**) were incubated with or without lambda protein phosphatase enzyme prior to western blot as before. Both mouse and *Xenopus* Ascl1 are phosphorylated.

Two cell-stage embryos were unilaterally injected with 100pg mRNA encoding either mAscl1 or xAscl1 and with βgal as a lineage tracer. At stage 18, embryos were assayed by *in situ* hybridisation (ISH) for expression of N-β-tubulin as a marker of primary neurogenesis ^19^, and for xMyt1 that is induced downstream of proneural proteins and mediates resistance to lateral inhibition ^17^ ( Figure 1A-C). xMyt1 expression mirrors N-β-tubulin expression, consistent with both of these genes being induced by the Ascl1 proteins. In comparison, mouse Ascl1 produces a moderate increase in expression of N-β-tubulin and xMyT1 across the lateral epidermis, while the *Xenopus* protein results in less pronounced ectopic neurogenesis that is concentrated in the region of the developing neural tube. As these experiments involve protein translation *in vivo* from over-expressed coding sequence mRNA, the observed differences in Ascl1 activity must be mediated at a post-translational level, rather than differences in Ascl1 gene expression or mRNA processing.

### Mouse and *Xenopus* Ascl1 proteins are both phosphorylated and show similar protein stability

Proneural transcription factors are known to be unstable proteins, which are turned over rapidly by the ubiquitin-proteosome system ^20^. To explore potential species differences in protein accumulation/stability following mRNA expression, Ascl1 levels in whole embryo extracts were compared by western blot, probing for the HA-tag at the C terminus of each protein ( Figure 1D). Mouse Ascl1 migrates as a single band and at a higher molecular weight than xAscl1, in part expected from the extended N terminus in the mouse protein (see below). In contrast, *Xenopus* Ascl1 migrates as a broad or multiple bands that may represent various post-translationally modified isoforms. When the amount of mAscl1 and xAscl1 is measured relative to the tubulin loading controls in two independent experiments (shown), there are no significant differences in protein accumulation; 1.60 +/- 0.11 units for xAscl1 and 1.54 +/- 0.08 units for mAscl1, indicating that accumulation and stability are similar for the two proteins and cannot account for the differences in activity.

Multi-site phosphorylation has been shown to have a marked inhibitory effect on Ascl1 proneural activity ^21^. To determine whether there are differences in phosphorylation between mAscl1 and xAscl1, extracts from embryos expressing the two proteins were treated with lambda protein phosphatase prior to separation and western blot ( Figure 1E). The multiple bands of xAscl1 protein collapse to a single faster migrating band in the presence of phosphatase enzyme, suggesting that the multiple bands in the untreated samples represent xAscl1 phospho-forms. Similarly, the single band of the mouse protein also undergoes a prominent increase in migration after phosphatase treatment, indicating that mouse Ascl1 is also phosphorylated, and indeed may be more uniformly phosphorylated than the *Xenopus* protein. Thus both proteins accumulate to comparable levels following over-expression and both undergo phosphorylation, so these properties cannot account for the observed *in vivo* differences in activity. 

### Mouse and *Xenopus* proteins are highly conserved except for the N terminus

**Figure 2.**
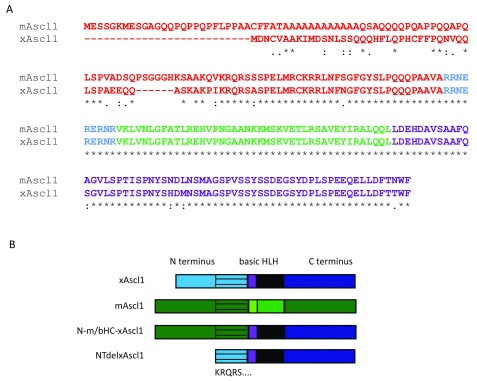
mAscl1 and xAscl1 proteins differ in their N terminal sequences. ( **A**) Protein sequence alignment of mAscl1 and xAscl1 using ClustalW software. N terminus shown in red; basic domain in blue; HLH domain in green; C terminus in purple. A consensus line is shown below the alignment to indicate the degree of conservation of amino acids at each position: (*) denotes identical residues; (:) denotes highly conserved residues; (.) denotes weakly conserved residues. ( **B**) Schematic representation of constructs made to investigate the importance of differences in the N terminus of the Ascl1 protein. The conserved region of the N terminus (from KRQRS) is shown with black stripes through the respective blue (xAscl1) or green (mAscl1) region, and this is retained in NTdelxAscl1 so that only the non-conserved region of the N terminus is deleted.

Protein sequence alignment of mAscl1 and xAscl1 reveals a high degree of species conservation in all but the N terminus ( Figure 2A). The bHLH domains of mouse and *Xenopus* proteins (shown in blue and green) are identical, and the C terminus (purple) differs by just four amino acids that are broadly conserved alternative residues. Furthermore, the 35 residues in N terminal fragment adjacent to the bHLH domain are identical in all but two residues between species. However, outside of this, the most N terminal regions of the two proteins diverge in both sequence and length.

To investigate the potential role of the N terminus in conferring Ascl1 protein activity, a chimeric construct was made ( Figure 2B) by substituting the non-conserved N terminal region of the *Xenopus* protein with the mouse equivalent (N-m/bHC-xAscl1). Similarly, a deletion mutant was made to remove this non-conserved N terminus from the *Xenopus* protein, leaving only the conserved regions from KRQRS… (NTdelxAscl1). 

### The non-conserved region of the N terminus mediates species differences in Ascl1 activity

**Figure 3.**
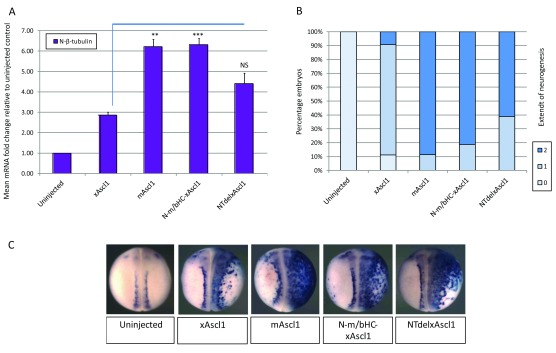
Differential activity between mAscl1 and xAscl1 maps to the N terminus of the protein. Two cell stage embryos were unilaterally injected with 100pg of mRNA encoding each construct, as labelled. At stage 18, embryos were assayed for expression of N-β-tubulin relative to uninjected control embryos. ( **A**) qPCR data [N=3] with significance calculated by paired student T test; NS = not significant; * = p< 0.05; ** = p< 0.025; *** = p< 0.0125. ( **B**) Semi-quantitative scoring of grade of neurogenesis after in situ hybridisation [N=49-75 embryos per category from two experiments]. ( **C**) Representative images of embryos with injected side to the right. The non-conserved N terminal region of mAscl1 is able to confer the activity of the mouse protein on chimeric xAscl1. Deletion of the non-conserved N terminus in xAscl1 also enhances the activity of xAscl1 but this deletion mutant is still not as active as mAscl1.

Using the *Xenopus* neurogenesis assay as before, mRNA encoding chimeric and truncated forms of Ascl1 ( Figure 2B) was injected unilaterally in two-cell stage embryos, and expression of N-β-Tubulin was detected in stage 18 embryos by qPCR and ISH ( Figure 3). Exchange of the non-conserved N terminal region of mouse Ascl1 in place of the shorter N-terminal region of xAscl1 results in enhanced activity of the chimera to the level of native mAscl1; by qPCR, both mAscl1 and N-m/bHC-xAscl1 induce around two-fold higher level of N-β-Tubulin expression than wild-type xAscl1. This indicates that the non-conserved region of the N terminus of the mouse protein mediates its relatively greater potency. Interestingly, deletion of the corresponding non-conserved region of the *Xenopus* protein without its replacement by the respective region from mAscl1 also increases N-β-Tubulin expression relative to wild-type xAscl1, although this is not significant on qPCR analysis. This may suggest that while the N terminus of the mouse protein enhances Ascl1 activity, the corresponding region of the *Xenopus* protein may have an inhibitory influence.

## Discussion/Conclusions

In this study we have determined that mouse Ascl1 protein is approximately twice as active as the *Xenopus* protein at inducing ectopic primary neurons in *Xenopus* embryos. This requires the presence of the non-conserved N terminal residues in the mouse protein, but is not mediated through changes in protein accumulation or Ascl1 phosphorylation.

Different activity between mouse and *Xenopus* homologues in *Xenopus* embryos has previously been reported for another master regulatory bHLH protein in muscle, MyoD. The C terminus of xMyoD causes cytoplasmic retention of the protein until mesoderm induction, while mMyoD is constitutively nuclear ^22^. The differing activity of mouse and *Xenopus* Ascl1 *in vivo* in *Xenopus* embryos may similarly be due to differences in nuclear localisation, although we have yet to explore this possibility. Species differences in activity could also be due to cofactor binding involving the N terminus of the protein, for instance, association of mouse Ascl1 with an activating partner or reduced association with an inhibitory factor. As such, if using *Xenopus* to dissect the molecular mechanisms of proneural activity, consideration should be given to potential differences in activity between homologous proteins from different species.

## Data availability

Raw data files are available in Open Science Framework: The N terminus of Ascl1 underlies differing proneural activity of mouse and *Xenopus* Ascl1 proteins: https://doi.org/10.17605/OSF.IO/CN76S ^23^

See *Methods* section for description of data analysis and ISH scoring. Datasets presented are as follows:

- Fig1A_ISH and Fig1B_ISH: Semi-quantitative scoring of expression of N-β-Tubulin (Fig1A) or xMyt1 (Fig1B) for each injection category showing total number of embryos from two independent experiments.
- Fig1C_embryos: Representative images from 50–78 embryos in each category in two independent experiments.
- Fig1D_WesternBlot.
- Fig1E_WesternBlot.
- Fig3A_qPCR: Mean fold change in N-β-tubulin expression relative to uninjected controls; three independent experiments.
- Fig3B_ISH: Semi-quantitative scoring of grade of neurogenesis for each injection category showing total number of embryos from two independent experiments.
- Fig3C_embryos: Representative images from 49-75 embryos in each category in two independent experiments.

Data are available under the terms of the Creative Commons Zero "No rights reserved" data waiver (CC0 1.0 Public domain dedication).
