# Supplementary material for: The N terminus of Ascl1 underlies differing proneural activity of mouse and Xenopus Ascl1 proteins
Source: Wellcome Open Res. 2018 Sep 26;3:125. [Version 1] doi: 10.12688/wellcomeopenres.14842.1 (PMC6182678; doi:10.12688/wellcomeopenres.14842.1)
Supplement: Peer review history

# Peer review: Referee response for version 1

**DOI:** 10.21956/wellcomeopenres.16171.r33989

**Published:** 2018-10-11

**Authors:** Muriel Perron

**Affiliations:** 1 Paris-Saclay Institute of Neuroscience, CNRS (French National Center for Scientific Research), University of Paris-Sud, University of Paris-Saclay, Orsay, France

In this research note, the authors have compared the proneural activity of *Xenopus* and mouse transcription factor Ascl1, by mRNA injection in *Xenopus* embryos. They demonstrated that the mouse Ascl1 protein is surprisingly more active in inducing ectopic neurons than the *Xenopus* one. Using chimeric and truncated forms of Ascl1, the authors determined that this species difference in Ascl1 activity is mediated by the N terminus of Ascl1. This is interesting considering that this region was identified as poorly conserved between the two species. The molecular mechanisms underlying its distinct influence in *Xenopus* and mouse remain to be investigated.

 Overall, the research note is clearly written, the methodology is sound and the data are convincing. The illustration in Fig. 1C could however be improved. It is explained that *Xenopus* Ascl1 mRNA injection leads to ectopic neurogenesis that is concentrated in the region of the developing neural tube (compared to broader ectopic neurogenesis observed following injection of the mouse mRNA). The figure indeed shows an embryo injected with *Xenopus* Ascl1 mRNA, displaying restricted ectopic N-tubulin expression. However, it also has a restricted ß-gal staining to the same region. It would have been more convincing to show an embryo with ß-gal staining on the whole side of the embryo and ectopic N-tubulin expression restricted to the neural tube region.

 Although the data appear convincing in graphs of Fig. 1A,B and Fig. 3B, statistical tests would have been appropriate to draw the conclusions.

 The authors wrote that Ascl1 is a central driver of neuronal reprogramming *in vitro*. Specifying *in vitro* is not necessary since it has also been shown *in vivo* that forced Ascl1 expression can trigger reprogramming, for instance in Müller cells in response to injury. The authors could cite Ueki *et al*., PNAS 2015.

 On western blots, it should be written alpha-tubulin since it could be confusing with the N-tubulin expression that is analyzed elsewhere in the manuscript.

 The authors determined that mouse Ascl1 protein is approximately twice as active as the *Xenopus* protein at inducing ectopic primary neurons in *Xenopus* embryos. It is not clear how this two-fold difference has been calculated.

I have read this submission. I believe that I have an appropriate level of expertise to confirm that it is of an acceptable scientific standard.

# Peer review: Referee response for version 1

**DOI:** 10.21956/wellcomeopenres.16171.r33987

**Published:** 2018-10-05

**Authors:** Colin Sharpe

**Affiliations:** 1 Institute of Biomedical and Biomolecular Science (IBBS), School of Biological Sciences, University of Portsmouth, Portsmouth, UK

In this Research Note, Hardwick and Philpott use a simple assay to assess the ability of mouse and Xenopus Ascl1 to generate primary neurones in Xenopus.  It is already known that Xenopus Ascl1 mRNA injected into an early stage Xenopus embryo will cause an increase in neurogenesis, the interesting result here is that the mouse version, injected into Xenopus, is even more active in this process.  By using a chimeric mouse-Xenopus Ascl1 and a deleted version of the Xenopus protein they provide evidence that the poorly conserved N-terminal part of the protein is responsible for differences in its activity.

 The report is concise and informative and the approach technically sound.  The results clearly implicate the N-terminal region of the proteins.  Other factors are excluded, since experiments on the broad phosphorylation status of the proteins indicate both are to some degree phosphorylated within the embryo and there are no significant differences in the accumulation of the Ascl1 proteins, suggesting that they have similar stabilities.

 The inclusion of two additional constructs, a truncated version of the mouse protein and a chimeric form with the Xenopus N-terminus in front of the rest of the mouse protein, would have provided a more complete comparison. The results as they stand, however, give a clear indication of the significance of the N-terminal region, which is the main aim of this short report, even if the underlying molecular mechanism remains uncertain. 

 As a minor point, a comment in the Methods section that the construction and cloning of the deleted version places a start codon directly adjacent to the remainder of the protein (KRQS...) might avoid some confusion.

 One aspect that could be covered in a more detail concerns the lack of conservation in the N-terminal region of Ascl1. It raises several questions:  How different is the sequence between the model organisms Xenopus, zebrafish, chicks and mice, for example and how conserved is the N-terminal region between two mammals such as mice and humans? Adding a brief comment in the text or a few more lines to the Clustal alignment to make the level of conservation more apparent would be helpful and would clarify whether Xenopus (or mouse) is an odd case, or whether this sequence just isn’t well conserved across the vertebrates.

I have read this submission. I believe that I have an appropriate level of expertise to confirm that it is of an acceptable scientific standard.
